# Neurological manifestations of COVID-19 infection: an umbrella review

**DOI:** 10.1186/s41983-021-00366-5

**Published:** 2021-08-28

**Authors:** Samad Shams Vahdati, Alireza Ala, Dara Rahmanpour, Elyar Sadeghi-Hokmabadi, Fateme Tahmasbi

**Affiliations:** 1 Emergency Medicine Research Team, Emergency Department, Tabriz University of Medical Sciences, Tabriz, Iran; 2 Student Research Committee, Tabriz University of Medical Sciences, Tabriz, Iran; 3 Neurosciences Research Center, Neurology Department, Tabriz University of Medical Sciences, Tabriz, Iran

**Keywords:** Corona virus disease 2019, COVID-19, Neurological manifestations, Neurology, Systematic review

## Abstract

**Background:**

Neurological involvements of COVID-19 are one of the most reported manifestations of this infection. This study aims to systematically review the previous systematic reviews which addressed the neurological manifestations of the COVID-19 infection.

**Methods:**

Following the Preferred Reporting Items for Systematic Review and Meta-Analysis (PRISMA) guidelines, a comprehensive search was conducted in PubMed, Embase, Scopus, Web of Science databases and Google Scholar from December 2019 to December 2020. Articles were critically screened by two independent reviewers and if met the inclusion criteria, entered the study. Assessment of methodological quality was conducted by Assessment of Multiple Systematic Reviews-2 (AMSTAR-2) tool. Statistical analysis was not applicable. From a total of 1302 studies, 308 studies were removed due to their irrelevant title and abstract. After screening the full texts, a total of 66 found to be eligible. Twenty-one studies reported general manifestations of the COVID-19, 13 studies reported cerebrovascular events, 19 olfactory and oral dysfunctions, 5 systematic reviews on Guillen–Barré syndrome (GBS) and 8 articles on the sporadic manifestations like ocular signs and symptoms. The majority of the studies were classified as critically low or low in terms of quality.

**Conclusion:**

Despite great heterogeneity in the current literature, neurological involvements are an important extra-pulmonary aspect of the COVID-19; most commonly in the form of general manifestations like headache and olfactory disturbances. Long-term effects of this virus on the nervous system must be a research priority for future references.

**Supplementary Information:**

The online version contains supplementary material available at 10.1186/s41983-021-00366-5.

## Introduction

In December 2019, coronavirus disease 2019 (COVID-19) started by an outbreak in China and soon afterwards, infected millions of people all over the world and revolutionized our whole perspective of the healthcare.

Most reported signs and symptoms of COVID-19 infection are fever, dry coughs and fatigue [1]. Nevertheless, respiratory invasions are not the only medical concern regarding this virus. According to many reports, COVID-19 has proven to be a multi-organ disorder with multiple extra-pulmonary manifestations, including cardiovascular, renal, gastrointestinal and neurological [2, 3].

The spectrum of neurological involvement of COVID-19 has been a growing body of literature, ranging from simple headaches to more severe manifestations like stroke and seizures [4–6]. Considering the immediate need of evidence, there are a great number of studies published every day. It is obvious that reaching a reliable and valid source of evidence is essential for clinical decision-making and on higher levels, policy-making. Therefore, a challenging field has come into the spotlight.

A systematic review on systematic reviews, also known as an umbrella review, is a type of novel methodology which aims to summarize an extensive scope of literature and provide a holistic view on a specific matter. Umbrella reviews can provide the highest level of evidence and benefit both clinicians and policy-makers [7, 8].

To our knowledge, no publications have reported this gap. Therefore, we conducted a systematic review on the previous systematic reviews that addressed the neurological signs and symptoms of the COVID-19 infection in an attempt to classify and broaden the current literature on the matter.

## Methods

This study is conducted in accordance to Preferred Reporting Items for Systematic Reviews and Meta-analysis (PRISMA) guidelines [9].

### Protocol and registration

The protocol of this study is registered and approved by the Research Ethics Committee of Tabriz University of Medical Sciences. (ID: IR.TBZMED.REC.1399.984) Written consent from patients was exempted because the population of this study is previously published documents.

### Eligibility criteria

Due to the extent of the academic literature on this matter, specific inclusion criteria was defined and applied; all the systematic reviews, with or without meta-analysis, which were conducted on the neurological manifestations of the SARS-CoV-2 infection from December 2019 to December 2020 was included in this umbrella review.

Exclusion criteria were as followed: (1) all the other methodology; including experimental studies, case-based studies, retrospective or prospective studies and narrative reviews; (2) studies with no report on the neurological signs and symptoms; (3) studies focused on a specific population like pregnant women, children, patients with specific conditions like cancer; (4) incomplete studies or studies with unavailable full text; (5) non-English studies and (6) animal studies.

### Information sources and search strategy

A comprehensive literature search was conducted in PubMed, Embase, Scopus Web of Science and Google Scholar form December 2019 to December 2020 to identify all the relevant articles. Related keywords to the COVID-19 and neurological manifestations, including “COVID-19”, “coronavirus”, “SARS”, “SARS-CoV-2”, “neurology”, “neurologic”, “neurologic manifestations”, “neurologic signs”, “neurologic symptoms”, “olfactory”, “anosmia”, “dysgeusia”, “stroke”, “cerebrovascular event”. “Guillen-barre syndrome”, “systematic review”, “meta-analysis” and other relevant synonyms and their combination with the proper Boolean operators. The search results were imported in the reference managing software EndNote X8 for further analysis.

### Study selection process

After the removing duplicates, the title and abstracts of the imported articles were evaluated by two independent reviewers (F.T and S.S.V). The remaining articles were then assessed through their full-text. Any sort of disagreement was dissolved by referring to a third reviewer (A.A).

### Assessing the quality of the systematic reviews

Assessment of methodological quality of included studies was conducted by Assessment of Multiple Systematic Reviews-2 (AMSTAR-2) tool [10]. This tool consists of 16 questions regarding the mythological strengths of systematic review and categorizes their quality as critically low, low, moderate and high. The form is attached in Additional file 1.

### Data extraction

The full texts of the included articles were carefully read and analyzed by two independent reviewers (F.T and S.S.V) in an Excel worksheet and following items were extracted: the name of the author, year of publication, country in which studies were conducted, the quality assessment according to AMSTAR-2 tool, population, types and number of the included studies, searched databases, neurological manifestations, the method of assessing the quality of included studies and main results. Any controversies among two reviewers were dissolved by referring to a third reviewer (A.A).

### Synthesis of results

Considering the heterogeneity of the systematic reviews and studies that each review included, statistical analysis was neither feasible nor appropriate; therefore a narrative synthesis of the results was conducted.

## Results

### Study selection

**Fig. 1:**
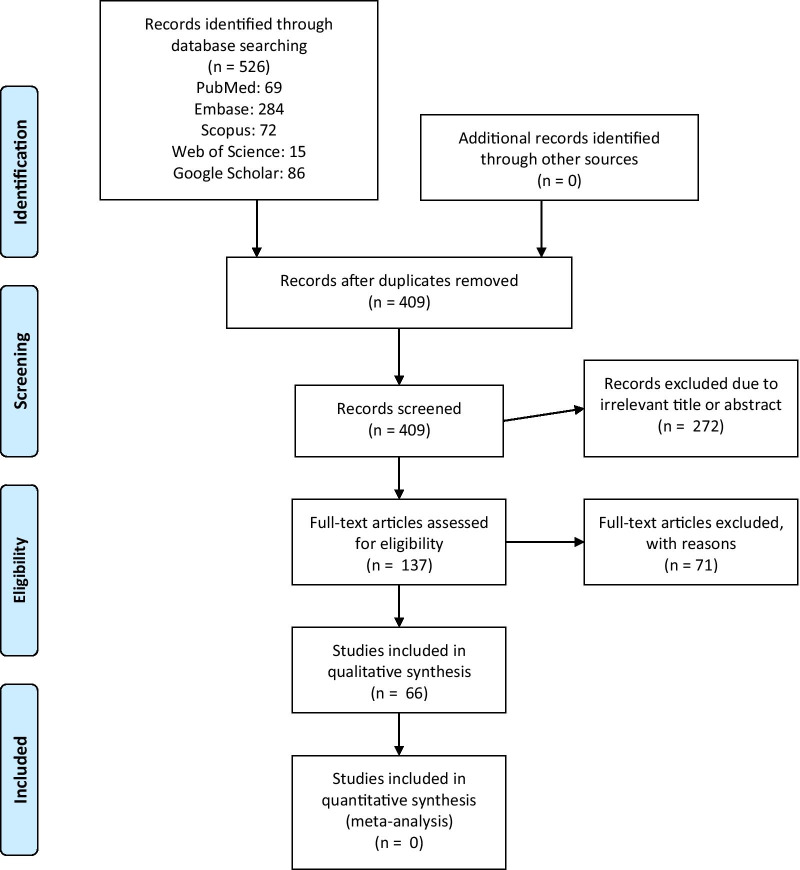
Search results and selection of studies for systematic review according to the PRISMA flowchart

**Fig. 2:**
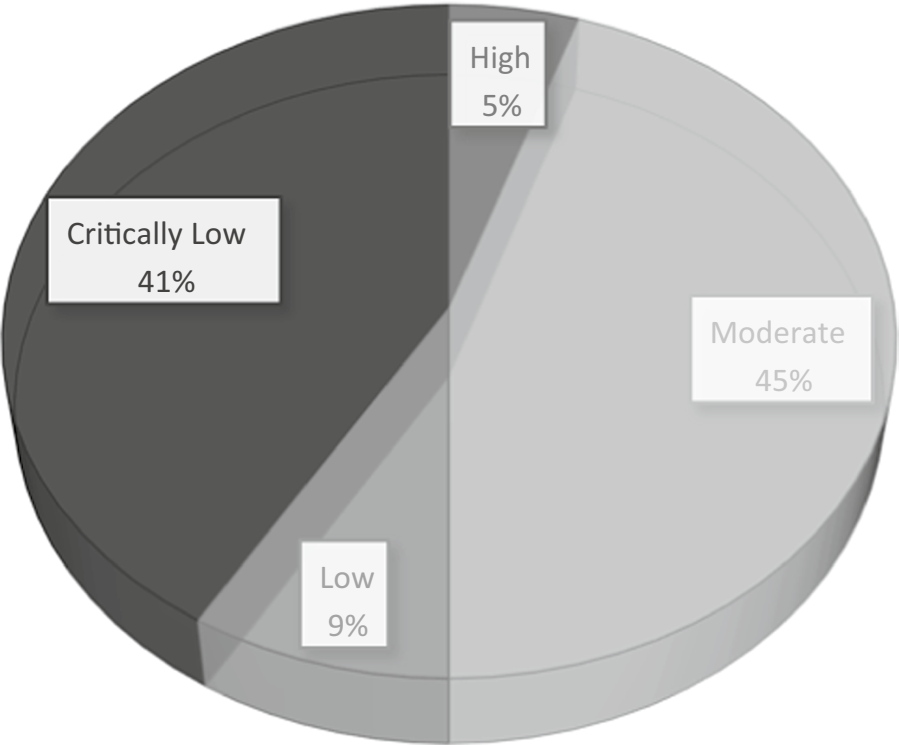
The quality of included studies according to the AMSTAR-2

From a total of 1302 studies, 476 studies remained after removing the duplicates. After screening the title and abstracts, 308 studies were removed due to their irrelevance, leaving 101 studies for the full-text screening. At last, a total of 66 studies were included in this systematic review. The process is summarized in Fig. 1. In addition, the results of quality assessment using the AMSTAR-2 tool are also presented in Fig. 2.

### Studies on general neurological manifestations

A total of 21 studies reported neurological findings in COVID-19 patients. Nine studies conducted a meta-analysis of the included studies [11–18]. Regarding the origin of the studies, seven studies were conducted in Europe [19–25], three in Iran [15, 26, 27] and one in each of the following countries China [18], Nepal [28], Nigeria [11], Philippines [13], India [14], Brazil [29], Indonesia [16], Singapore [12], Taiwan [17], Egypt [30], USA [31]. The number of their included studies varied from 7 to 212. Population of the studies consisted of patients with different stages of COVID-19 infection.

Different studies included a wide spectrum of neurological manifestations; some studies included muscular or musculoskeletal involvement [11, 28, 31], several studies included cerebrovascular events [11, 12, 15–19, 21, 22, 24–29, 31]; several studies cover olfactory and gustatory dysfunctions or OGD [11, 13, 14, 17, 18, 20–26, 28, 29], dizziness was mentioned in eight studies [11, 13, 14, 16, 17, 19, 21, 30], inflammation of the brain tissue or adjacent structure like meninges was mentioned in five studies [12, 18, 23, 29, 31], impaired concussion also mentioned in five studies [11, 13, 16, 19, 20].

According to one study, the most common neurological manifestations in hospitalized patients were headache and anosmia [25]. In several studies, headache continued to be described as the most common neurologic sign in COVID-19 patients [13, 19, 20, 26, 28, 30]. It was also mentioned by other studies with different prevalence [11, 14, 16, 17, 21]. One study stated that stroke is the most frequent type of neurological dysfunction in COVID-19 patients with the highest mortality rate [12]. One study reported muscle injury or myalgia as the most common type of neurological disturbance [16]. Another study reported that fatigue is the most common non-specific involvement of the nervous system, alongside the anorexia, dyspnea/shortness of breath, and malaise [18]. OGD was also stated as the most common type of neurologic involvement by five studies [11, 14, 17, 22, 24].

Further information is presented in Table 1.

**Table 1:** The characteristics of included systematic reviews regarding the general manifestations of COVID-19 infection

Authors	Origin	Type	Quality	Number of included studies	Types of included studies	Assessing the quality of included studies	Searched databases	Main results
1. Whittaker et al. 2020 [25]	UK	Systematic review	Critically Low	31	Cohort, case report, cross-sectional, case–control	None	Pubmed, Embase, Scopus, Google Scholar, Cochrane Library, Ovid	Headache and anosmia were the most common neurological manifestations of SARS-CoV-2 in hospitalized patients. Less common symptoms include seizure, stroke and isolated cases of GBS^1^
2. Wang et al. 2020 [18]	China	Systematic review + meta-analysis	Critically Low	41	Case series, case report, cross-sectional, case–control, letters	NIH^2^ quality assessment tool	Pubmed, Embase, Scopus, medRxiv, bioRvix	The most common manifestationswere fatigue, anorexia, dyspnea/shortness of breath, and malaise. The common specific neurological symptoms included OGD^3^, especially in mild cases. GBS and acute inflammation of the brain, spinal cord, and meninges
3. Chen et al. 2020 [20]	Germany	Systematic review	Moderate	92	Cohort, case series, case report, case–control, letters	1. Criteria for diagnosis of Covid-192. Laboratory confirmation method 3. The respiratory specimens used for testing	Pubmed, medRxiv, bioRxiv, Three Chinese databases	Headache, dizziness, taste and smell dysfunctions, and impaired consciousness were the most frequently described neurological symptoms
4. Neishaboori et al. 2020 [15]	Iran	Systematic review + meta-analysis	Moderate	7	Cohort, case series, case–control	NHLBI^4^ quality assessment tool	Pubmed, Embase, Scopus, Web of Science	The most common type of CNS^5^ complications included encephalopathy and acute cerebrovascular disease
5. Di Carlo et al. 2020 [21]	Italy	Systematic review	Moderate	19	Cohort, case series, case report, case–control	Modified NOS^6^	Pubmed, Embase	Headache, dizziness, OGD were reported in patients, three studies reported radiological confirmed acute cerebrovascular disease
6. Asadi-Pooya et al. 2020 [26]	Iran	Systematic review	Critically Low	8	Cohort, case series, case report	None	Pubmed, Scopus	Nonspecific neurological symptoms, such as confusion and headache were reported in COVID-19 patients. A few patients also showed more specific manifestations, such as seizure or cerebrovascular problems
7. Nepal et al. 2020 [28]	Nepal	Systematic review	Critically Low	37	Cohort, case series, case report, case–control	None	Pubmed, Google Scholar, Chinese National Knowledge Infrastructure, Research square, medRxiv, Social Science Research Network, and ChinaXiv	The most commonly reported neurological manifestations of COVID-19 weremyalgia, headache, altered sensorium and OGD. Uncommonly, COVID-19 can also present with CNS manifestations such as ischemic stroke, intracerebral hemorrhage, encephalo-myelitis, and acute myelitis, PNS^7^ manifestations such as GBS and Bell’s palsy, and skeletal muscle manifestations such as rhabdomyolysis are also reported
8. Taherifard et al. 2020 [27]	Iran	Systematic review	Critically Low	22	Case series, case report	None	Pubmed, Scopus, Web of Science	The virus seemed to affect both PNS and CNS. The most frequently reported neurological complication was acute ischemic cerebrovascular accident, followed by GBS syndrome
9. Ghannam et al. 2020 [31]	USA	Systematic review	Moderate	42	Case series, case report	JBI^8^ Critical Appraisal Tool	Pubmed, Ovid Medline	COVID-19 patients demonstrated cerebrovascular insults, neuromuscular disorders, and encephalitis or encephalopathy
10. Abdullahi et al. 2020 [11]	Nigeria	Systematic review + meta-analysis	Moderate	60	Cohort, case series, case report, cross-sectional, case–control	Modified McMaster critical review form	Pubmed, Embase, Google Scholar, Web of Science	The most common type of neurological and musculoskeletal manifestations were as followed: OGD, myalgia, headache, back pain, dizziness, acute cerebrovascular disease and impaired consciousness
11. Cagnazzo et al. 2020 [19]	France	Systematic review	Moderate	39	Case series, case–control	Modified NOS	Pubmed, Embase, Scopus	Headache, muscle injury, psychiatric involvement, impaired consciousness, OGD, acute cerebrovascular events and dizziness were the most frequently reported neurological manifestations. Less common ones were ischemic stroke, cranial nerve impairment, nerve root and plexus disorders, epilepsy, and hemorrhagic stroke
12. Collantes et al. 2020 [13]	Philippines	Systematic review + meta-analysis	High	35	Cohort, case series, case report, case–control	Murad tool	Pubmed, Embase, Scopus, WHO database	The most common type of neurological involvements were headache, dizziness, headache and dizziness, nausea, vomiting, nausea and vomiting, confusion and myalgia. The most common neurological complication associated with COVID-19 was vascular disorders; others included encephalopathy, encephalitis, oculomotor nerve palsy, isolated sudden-onset anosmia, GBS, and Miller–Fisher syndrome
13. Orrù et al. 2020 [22]	Italy	Systematic review	Critically Low	87	Cohort, case report, case–control, in-press articles	None	Pubmed, Scopus	OGD, ischemic/hemorrhagic stroke, meningoencephalitis and encephalopathy, including acute necrotizing encephalopathy were commonly associated with COVID-19; PNS involvements included, GBS and Miller Fisher syndromes
14. Favas et al. 2020 [14]	India	Systematic review + meta-analysis	High	212	Cohort, experimental, case report, cross-sectional, case–control, letters	NOS, CONSORT^9^	Pubmed, Embase, Scopus, Google Scholar, Cochrane Library, Web of Science, WHO database, EBSCO, Clinicaltrial.gov	OGD, myalgia, headache, dizziness, and syncope are reported in COVID-19. Ischemic stroke, followed by hemorrhagic stroke, and cerebral venous thrombosis were also reported
15. Munhoz et al. 2020 [29]	Brazil	Systematic review	Critically Low	43	Cohort, experimental, case series, case report, case–control	None	Pubmed, LILACS	Non-specific symptoms, such as hyposmia and myalgia, to more complex and life-threatening conditions, such as cerebrovascular diseases, encephalopathies, and GBS are associated with COVID-19 infection
16. Romoli et al. 2020 [23]	Italy, Austria, Zurich, Switzerland	Systematic review	Moderate	27	Cohort, case series, case report, cross-sectional, case–control	NOS	Pubmed, Embase, Google Scholar, medRxiv, ChinaXiv	Cases of OGD, GBS, Miller-Fisher syndrome, cranial neuropathy, meningitis, encephalitis, and various other conditions were linked to COVID-19
17. Pinzon et al. 2020 [16]	Indonesia	Systematic review + meta-analysis	Moderate	33	Cohort, experimental, case report, ross-sectional, case–control	The Oxford CEBM^12^	Pubmed	Myalgia was the most common, neurologic symptom of COVID-19, followed by headache, dizziness; nausea with or without vomiting; concurrent cerebrovascular disease; and impaired consciousness
18. Chua et al. 2020 [12]	Singapore	Systematic review + meta-analysis	Critically Low	48	Cohort, case series, case report, cross-sectional, case–control, letters, rapid comments	None	Pubmed, Google Scholar	Stroke is the most frequently reported neurological manifestation in COVID-19 and has the highest mortality rate. Other manifestations included GBS and variants, meningitis, encephalitis, encephalopathy, myelitis and seizures
19. Scoppettuolo et al. 2020 [24]	Switzerland, Belgium	Systematic review	Critically Low	42	Case series, case report	None	Pubmed, Scopus, Cochrane Library	Neurological complications of SARS-CoV-2 are mainly related to OGD, cerebrovascular disease and PINS^10^ are rare
20. Tsai et al. 2020 [17]	Taiwan	Systematic review + meta-analysis	Critically Low	79	Case series, case report	None	Pubmed, Embase, Cochrane Library	OGD, myalgia, headache, acute cerebral vascular disease, dizziness, altered mental status, seizure, encephalitis, neuralgia, ataxia, GBS, Miller Fisher syndrome, intracerebral hemorrhage, polyneuritis cranialis, and dystonic posture were demonstrated in COVID-19 patients
21. Ibrahim 2020 [30]	Egypt	Systematic review	Critically Low	20	Cohort, experimental, case series, case report, cross-sectional	None	Pubmed, Scopus, Cochrane Library, clinicaltrials.gov	CNS symptoms was more common compared to the PNS. Headache was the most common neurological symptoms in regard to number of patients, meanwhile dizziness had the highest incidence

1. *GBS:* Guillain–Barré syndrome, 2. *NIH:* National Institutes of Health, 3. *OGD:* Olfactory and Gustatory Disorder, 4. *NHLBI*: National Heart, Lung, and Blood Institute, 5. *CNS:* central nervous system, 6. *NOS:* Newcastle–Ottawa Scale, 7. *PNS:* Peripheral Nervous System, 8. *JBI:* Joanna Briggs Institute, 9. *CONSORT:* Consolidated Standards of Reporting Trials, 10. *PINS:* Post-infection Neurological Syndrome. P value < 0.05 was considered to be statistically significant

### Studies on cerebrovascular accidents

A total of 13 studies were included in this systematic reviews. Six studies conducted meta-analysis [32–37], one study was a cohort plus a systematic review [38] and one study was the combination of a narrative review and systematic one [39]. Five studies were conducted in Europe [34, 38–40], two in USA [35, 41], and one in each following countries; Colombia [42], South Korea [43], Malaysia [32], India [44], Canada [33] and Singapore [37]. The population of each study was the COVID-19 patients with a form of cerebrovascular accident and different forms of stroke. Two studies did not report the type of articles included in the study [33, 43].

The severity of the infection was mentioned several times across studies, stating the fact that the more severe the infection becomes, the higher the risk of cerebrovascular events, including strokes [32, 36, 38, 40].

The onset of the stroke was evaluated by two studies; one provided evidence supporting a possible trend between the severity of the COVID-19 infection and the temporality of stroke; meaning that mild infection is associated with early onset and severe infection is associated with late-onset stroke [42]. This evidence was supported by another study which suggested the Neutrophil–Lymphocyte Ratio (NLR) before hospitalization is positively correlated to the duration of the features of acute ischemic stroke (AIS) [40].

The mortality rates among these patients were also assessed by several studies, one study reported that COVID-19 infection is associated with higher mortality rates in stroke patients [44]. The other study indicated that the mortality of stroke in COVID-19 patients is associated with the age, comorbidities and the severity of the infection as stated [33]. Furthermore, one study suggested that stroke can be a prognostic factor and an indicator of the severity of the infection [32].

Concurring the aforementioned findings, another study stated that COVID-19 infection is associated with an increased risk of ischemic stroke, especially cryptogenic type; in addition to an increased risk of mortality [34]. While two studies mentioned ischemic stroke as the most common type of stroke among infected patients [39, 43]. A number of studies investigated the possible risks which were associated with stroke occurrence in COVID-19 infection—age, presence of other comorbidities or stroke risk factors including a history of cerebrovascular disorders, hypertension, hyperlipidemia or diabetes [33, 36, 41].

Additional information of these studies is provided in Table 2.

**Table 2:** Characteristics of included systematic reviews regarding stroke in COVID-19 infection

Authors	Origin	Type	Quality	Number of included studies	Types of included studies	Assessing the quality of included studies	Searched databases	Main results
1. Valencia-Enciso et al. 2020 [42]	Colombia	Systematic review	Moderate	47	Cohort, case series, case report, case–control	NOS	Pubmed, Scopus	A positive correlation seemed to exist between COVID-19 severity and temporality of stroke
2. Fraiman et al. 2020 [43]	South Korea	Systematic review	Critically Low	80	Not mentioned	None	Pubmed	Cerebrovascular events, especially ischemic stroke, were a common neurological manifestation in COVID-19 patients
3. Lee et al. 2020 [32]	Malaysia	Systematic review + meta-analysis	Moderate	28	Cohort, case series, case report	STROBE^2^	Pubmed, Medline, Cinhal	Stroke is an uncommon symptom in COVID-19 patients, but can be prognostic factor and an indicator of the severity of the infection
4. Bhatia et al. 2020 [44]	India	Systematic review	Moderate	30	Cohort, case series, case report	Oxford CEBM^3^ critical appraisal tool	Pubmed, Embase, Scopus	COVID-19 is associated with higher mortality rates in stroke patients
5. Wijeratne et al. 2020 [40]	Australia	Systematic review	Low	18	Cohort, case series, case report, case–control, reviews	None	Pubmed, Embase, Cochrane Library, Medline, Cinhal, Ovid	Neutrophil–Lymphocyte ratio at time admission is associated with the duration before onset of clinical features of AIS^3^
6. Fridman et al. 2020 [33]	Canada	Systematic review + meta-analysis	Low	10	Not mentioned	None	Pubmed, medRxiv, bioRxiv, Research Square search engines	The mortality of Stroke in COVID-19 patients is associated with age, comorbidities and the severity of the infection
7. Yamakawa et al. 2020 [35]	USA	Systematic review + meta-analysis	Moderate	26	Cohort, case series, case report, case–control	Assessment of risk of bias in prevalence studies	Pubmed, Embase,	The frequency of detected stroke in hospitalized patients was associated with age and other stroke risk factors
8. Katsanos et al. [34]	Greece	Systematic review + meta-analysis	High	18	Cohort	NOS	Pubmed, Scopus	COVID-19 infection is associated with an increased risk of ischemic stroke, especially cryptogenic stroke; in addition to an increased risk of mortality
9. Tan et al. 2020 [37]	Singapore	Systematic review + meta-analysis	Moderate	39	Cohort, case series, case report	NOS, JBI^5^ tool	Pubmed, Embase	AIS is associated with COVID-19 infection with a high mortality rate
10. Nannoni et al. 2020 [36]	UK	Systematic review + meta-analysis	Moderate	61	Cohort, case series, case report, case–control, letters	NOS	Pubmed, Scopus, MedRxiv	Acute cerebrovascular events are associated with the severity of the disease and pre-existing vascular risk factors in COVID-19 patients
11. Fatima et al. 2020 [41]	USA	Systematic review	Moderate	6	Cohort, case series, case report	GRADE^6^, Cochrane Collaboration’s tool	Pubmed, Embase, Scopus, Google Scholar, Cochrane Library, Medline	Stroke is associated with COVID-19 infection in patients with underlying risk factors including hypertension
12. Siepmann et al. 2021 [38]	Germany	Cohort + Systematic review	Critically Low	2	Cohort, experimental	Oxford CEBM tool	Pubmed, Embase, Cochrane Library	The severity of COVID-19 infection is associated with an increased risk of acute stroke
13. Szegedi et al. 2020 [39]	Germany	Narrative review + Systematic review	Critically Low	25	Cohort, case series, case report	None	Pubmed, Scopus	In COVID-19 patients the most common type of stroke was AIS

1. *NOS* :Newcastle–Ottawa Scale, 2. *STROBE:* Strengthening the Reporting of Observational Studies in Epidemiology, 3. *CEBM:* Centre for Evidence-Based Medicine, 4. *AIS:* Acute Ischemic Stroke, 5. JBI: Joanna Briggs Institute 6. *GRADE:* Grading of Recommendations Assessment, Development and Evaluation. P value < 0.05 was considered to be statistically significant

### Studies on olfactory and oral dysfunction

A total of 19 systematic reviews were included regarding olfactory and gustatory dysfunction (OGD) in COVID-19 patients with different severity of the infection, but in some cases the diagnosis of COVID-19 was not finalized. In addition, the population of one study was consisted of health-care workers [45]. Regarding the methodology, meta-analysis was conducted in 10 studies [46–55] and one study was a cohort plus a systematic review of literature [56]. The origins of the studies were as followed: four in UK [45–47, 57], three in Italy [54, 58, 59], two in USA [49, 60], two in China [53, 56] and one in each Brazil [61], United Arab Emirates [62], Thailand [48], Nigeria [55], Iran [52], Australia [50], Singapore [51] and Greece [63].

All of the included studies agreed on the positive correlation of the OGD and COVID-19 infection. One study suggested that the olfactory dysfunction might be the only COVID-19 infection in some cases [56]. The other studies provided evidence for the possible fact that the OGD might be the very first manifestation of the infection [53, 59, 60].

One study exclusively stated that OGD has a higher prevalence in female and younger patients [57]. Moreover, one study suggested that the presence of other respiratory infections might lead to more severe cases on COVID-19-related anosmia [63].

The relationship between the severity of the infection and OGD was not conclusively investigated by the included studies, but in some of them was reported as the secondary outcome; for instance, one study demonstrated that anosmia is more frequent in non-hospitalized COVID-19 patients than in hospitalized [54]. Furthermore, in one study OGD symptoms were reported in both ambulatory and hospitalized patients and mild-to-severe cases of COVID-19 patients [62]. Corroborating these statements, one study claimed that OGD should be considered as a clinical manifestation of the COVID-19 infection even in mild cases [61]. Finally, the other study evinced that OGD was estimated to be 31% and 67% in severe and mild-to-moderate symptomatic patients, respectively [46].

Most importantly, their included studies adopted widely different methods for diagnosis of OGD, varying from self-reporting [46, 49, 51, 53–55, 60–63], physician reporting [55, 60], online questionnaires [51, 55, 58], the University of Pennsylvania Smell Identification Test (UPSIT) [55–57, 60, 62], British version of UPSIT [45], Quality of life tool (sQOD‐NS) [47, 61], the “Sniffin Sticks” test [51, 55, 57], Subjective assessment using SNOT − 22 [51, 57], the Italian version of SNOT-22 [51], the Connecticut Chemosensory Clinical Research Center test (CCCRC) [48, 55, 57], COVID-19 Anosmia Reporting Tool for Clinicians [57, 63], standardized chemosensitive tests with odorants [58], non-validated questionnaires [58], butanol threshold assessment [48], case notes review (explicitly asked) [51], interviewing [51, 55, 62], history and physical examination [49, 54] and Smartphone based apps [55, 63]. Several articles did not report the method of OGD diagnosis among the included study [50, 52, 59].

Additional information of these studies is provided in Table 3.

**Table 3:** The characteristics of included systematic reviews regarding the olfactory and oral dysfunction of COVID-19 infection

Authors	Origin	Type	Quality	Number of included studies	Types of included studies	Assessing the quality of included studies	Searched databases	Main results
1. Borsetto et al. 2020 [46]	Critically Low	Systematic Review + Meta-analysis	UK	18	Not mentioned	None	Embase, Scopus, Web of Science, Medline, MedRxiv	The alteration of the sense of smell or taste was estimated 31% in severe and 67% in mild-to-moderate symptomatic patients
2. da Costa et al. 2020 [61]	Moderate	Systematic Review	Brazil	6	Cohort, cross sectional, case–control	NOS^1^	Pubmed, Scopus, Google Scholar, Cochrane library, LILACS^2^, Science direct	OGD^3^ occurs at varying intensities and prior to the general symptoms of COVID-19
3. Samaranayake et al. 2020 [62]	Moderate	Systematic Review	United Arab Emirates	8	Cohort, cross sectional, case–control	Nine-item checklist for prevalence study	Pubmed, Web of Science, EBSCO	Anosmia and dysgeusia symptoms were present in both ambulatory and hospitalized patients and mild-to-severe cases of COVID-19
4. Lechner et al. 2020 [45]	Critically Low	Systematic Review	UK	31	Case series, case reports, cross sectional	None	Pubmed	OGD is indicative of COVID-19 infection and should be implicated in evaluation of healthcare workers
5. Rocke et al. 2020 [47]	Moderate	Systematic Review + Meta-analysis	UK	12	Case series, cross sectional, case–control	ROBINS‐E	Pubmed, Embase, Cochrane library, HMIC, MedRxiv	There is a significant evidence demonstrating an association between olfactory dysfunction and COVID‐19
6. Zahra et al. 2020 [57]	Moderate	Systematic Review	UK	23	Cohort, case series, cross sectional, case–control	NOS	Pubmed, Scopus, Google Scholar, Cochrane library, Medline	Symptoms of anosmia and dysgeusia were frequently reported by COVID-19-positive patients; more commonly in females and in younger patients
7. Fuccillo et al. 2020 [58]	Moderate	Systematic Review	Italy	32	Cohort, case series, cross sectional	NHLBI^4^ Assessment Tools, Oxford CEBM^5^ guide	Pubmed, Embase, Web of Science	Olfactory disorders represent an important clinical characteristic of COVID-19
8. Hoang et al. 2020 [48]	Moderate	Systematic Review + Meta-analysis	Thailand	14	Case series, cross sectional, case–control	Modified NOS	Pubmed, Embase, Scopus, Medline OVID	OGD and COVID-19 are associated
9. Tong et al. 2020 [49]	Moderate	Systematic Review + Meta-analysis	USA	10	Cohort, case series, cross sectional, case–control	The quality assessment checklist for prevalence studies adapted from Hoy et al.	Pubmed, Scopus	OGS are common symptoms in patients with COVID-19 and may be manifested as an early symptoms in the clinical course of the COVID-19 infection
10. Passarelli et al. 2020 [59]	Critically Low	Systematic Review	Italy	5	Not mentioned	None	Pubmed	Anosmia and ageusia are a significant sign and can be considered as the first manifestation of the infection
11. Agyeman et al. 2020 [50]	Moderate	Systematic Review + Meta-analysis	Australia	24	Not mentioned	Murad tool	Pubmed, Embase, Medline, MedRxiv	High prevalence of OGD among patients infected with COVID-19 are reported across the literature
12. Pang et al. 2020 [51]	Moderate	Systematic Review + Meta-analysis	Singapore	19	Cohort, case series, cross sectional, case–control	The risk of bias tool for prevalence studies by Hoy et al.	Pubmed	Patient-reported olfactory dysfunction is a highly specific manifestation of COVID-19
13. Hajikhani et al. 2020 [52]	Moderate	Systematic Review + Meta-analysis	Iran	15	Not mentioned	JBI^6^ tool	Pubmed, Embase, Web of Science	OGD in patients with confirmed COVID-19 have a high prevalence
14. Sedaghat et al. 2020 [60]	Critically Low	Systematic Review	USA	6	Not mentioned	None	Pubmed, Embase, Web of Science	OGD is highly common in the course of COVID-19 infection and patients may experience sudden-onset of smell alteration as the first symptom
15. Chi et al. 2020 [53]	Low	Systematic Review + Meta-analysis	China	12	Not mentioned	None	Pubmed, Embase, Cochrane library, Cumulative Index to Nursing and Allied Health Literature, National Digital Library of Theses and Dissertations in Taiwan database, Art Image Indexing Service on the Internet Database (Chinese database)	OGD is associated with COVID-19 infection and in some patients is the first symptom of the infection
16. Giorli et al. 2020 [54]	Critically Low	Systematic Review + Meta-analysis	Italy	11	Cohort, cross sectional, case–control	None	Pubmed, Scopus, Web of Science	New onset olfactory dysfunction is associated with COVID-19, anosmia is more frequent in non-hospitalized COVID-19 patients than in hospitalized ones
17. Ibekwe et al. 2020 [55]	Moderate	Systematic Review + Meta-analysis	Nigeria	32	Cohort, cross sectional, case–control	JBI tool	Pubmed, Embase, Google Scholar, Web of Science	The prevalence of smell and taste loss among COVID-19 patients was high globally
18. Chung et al. 2020 [56]	Critically Low	Cohort + Systematic Review	China	23	Cohort, experimental, case series, case reports	None	Pubmed	COVID-19-related smell disturbance can be severe and prolonged and may be the only symptom
19. Printza et al. 2020 [63]	Critically Low	Systematic Review	Greece	24	Cohort, case series, cross sectional, case–control	None	Pubmed, Cochrane library, MedRxiv	Anosmia is more prevalent in COVID-19 patients than in patients suffering from other respiratory infections or controls

1. NOS: Newcastle–Ottawa Scale, 2. LILACS: Latin American and Caribbean Health Sciences Literature; 3. OGD: Olfactory and Gustatory Dysfunctions; 4. NHLBI: National Heart, Lung, and Blood Institute; 5. CEBM: Center for Evidence-Based Medicine; 6. JBI: Joanna Briggs Institute

### Studies on Guillen–Barré syndrome

A total of 5 systematic reviews were conducted on Guillen–Barré Syndrome (GBS) and reporting data from case series, case reports and one cross-sectional study**.** Three of these studies were conducted in Europe [64–66] one in Bangladesh [67] and one in Chile [68]. Only one study conducted meta-analysis [67]. Two of these systematic reviews also conducted a quality assessment for including the studies [67, 68]. The population of these studies consisted of COVID-19 patients, either with or without laboratory confirmed diagnosis, which showed GBS variants and subtypes manifestations. All of the included studies suggested a strong association between COVID-19-associated GBS to the classic GBS. One of the studies drew attention to the fact that the prognosis of COVID-19-associated GBS patients seems to worsen by increasing of age [66]. Additional data are provided in Table 4.

**Table 4:** The characteristics of the included systematic reviews regarding Guillen-Barre syndrome in COVID-19 infection

Authors	Origin	Type	Quality	Number of included studies	Types of included studies	Assessing the quality of included studies	Searched databases	Main results
1. Carrillo-Larco et al. 2020 [64]	Europe	Systematic review	Critically low	8	Case series, case report	None	Embase, Scopus, Web of Science, Global Health, Medline, MedRvix	Basic evidence suggests that GBS^1^ occurs after COVID-19 onset
2. Gittermann et al. 2020 [68]	Chile	Systematic review	Low	24	Case series, case report	NHLBI^2^ tool	Pubmed, Cochrane Library, Science Direct, Medline, WHO search tool	There seem to be a strong association between the GBS and Covid-19 which differs in presentations, including the severity of the GBS manifestations
3. Uncini et al. 2020 [65]	Europe	Systematic review	Critically low	33	Case series, case report	None	Pubmed	Classical GBS is presented in Covid-19 patients
4. Hasan et al. 2020 [67]	Bangladesh	Systematic review + meta-analysis	Low	45	Case series, case report, cross sectional	JBI^3^ tool	Pubmed, Web of Science, Cochrane Library, Web of Science	An association exists between classic GBS and Covid-19. These manifestations are responsive to GBS standard treatments
5. Abu-Rumeileh et al. 2020 [66]	Germany	Systematic review	Critically low	52	Case series, case report, reviews with case reports, reviews, letters, original article, point of view, and brief report	None	Pubmed, Google Scholar	COVID-19-associated GBS seems to share most features of classic post-infectious GBS with possibly the same immune-mediated pathogenetic mechanisms

1. GBS: Guillain-Barré Syndrome; 2. NHLBI: National Heart, Lung, and Blood Institute; 3. JBI: Joanna Briggs Institute

It is note-worthy that another systematic review had been conducted on the GBS and COVID-19, but it investigated how COVID-19 can affect previously diagnosed GBS patients. Hence, it was excluded from our study [69].

### Studies on sporadic types of neurological manifestations

Some studies reported sporadic neurological manifestations in COVID-19 patients; including two studies specifically on oral findings which reported dysgeusia [70, 71] and oral mucosal lesions, [71] in COVID-19 patients. Two studies were conducted on ocular manifestations; one study investigated the effects of prone positioning on ocular complications in COVID-19 patients in the critical care ward and reported that ocular surface disease, acute angle closure, ischemic optic neuropathy, orbital compartment syndrome and vascular occlusions are likely to occur in these patients [72]. The other study stated that ocular pain, discharge, redness and follicular conjunctivitis can arise from COVID-19 infection [73].

Auditory signs and symptoms was the main focus in one study; according to which in COVID-19-positive patients, hearing loss, tinnitus, and vertigo have rarely been reported [74].

ENT (Ear Nose Throat) manifestations were reported in one study that stated sore throat and headache as the most common ENT involvements in COVID-19 infection, while pharyngeal erythema, nasal congestion, rhinorrhea, upper respiratory tract infection, and tonsil enlargement were also reported with limited prevalence [75]. Furthermore, one study which aimed to assess the association between COVID-19 and encephalitis reported the occurrence of this complication in severe cases of infection and suggesting that COVID-19 virus can be considered as a neuropathogen with the ability to attack the nervous system regardless of respiratory symptoms [76]. At last, intracranial hemorrhage (IHC) was the subject in another systematic review. This study suggested that IHC is relatively uncommon among COVID-19 patients, but has a correlation with higher mortality rates [77].

Quality assessment was not conducted due the limited number of these studies.

## Discussion

The overall results of this umbrella review, which was conducted on 66 systematic reviews, demonstrated that neurological involvements are greatly common among COVID-19 patients with many different general, specific and sporadic manifestations.

The majority of the systematic reviews seemed to be low on methodological quality aspect. This fact can be justified by the outburst of the infection and an urgent need to access some evidence for clinical decisions and policy-making [78]. The results of the methodological assessment of our work is reminiscent of a previous study which was conducted to evaluate the quality of 49 systematic reviews regarding Severe Acute Respiratory Syndrome (SARS) and Middle East Respiratory Syndrome (MERS). This study indicated that the most systematic reviews regarding the coronavirus do not have satisfactory methodological quality [79].

Headache, as a general manifestation, was mentioned most commonly by studies. The characteristics of this headache are described as pressing, worsened by physical activities or head movements [80]. In another study, this headache was described to have a pulsating, pressing, and stabbing quality [81]. Different patterns of involvement are described including mostly holocranial [80, 82, 83], hemicranial or occipital [80] or bilateral and frontal [83]. Moreover, individuals with a migraine history are predisposed to have an earlier, longer and more severe headaches in comparison with migraine-free patients [83]. Preexisting primary headache was significantly related to the higher frequency of headache caused by COVID-19 infection [82], while one study mentioned that majority of its study population did not have a history of prior headaches [84]. Limited studies have addressed the associated risk factors. In one study, anosmia, myalgia, female sex and fever were reported as independent factors leading to a higher odds of headache in COVID-19 patients [85].

Regarding the sporadic manifestation of the disease, in addition to the conditions which were priorly mentioned, several neurological manifestations have been linked to the SARS-CoV-2; including seizures [86, 87], demyelination [88] and encephalopathies [87] and meningitis [89]. Many different types of viral infections can lead to such presentations, which makes it impossible to settle on the fact that COVID-19 infection is directly leading to these clinical signs and symptoms. For instance, both acute symptomatic seizures and late unprovoked seizures are reported in almost all types of acute central nervous system viral infections [90]. Another example is applied to meningitis which has been presented in multiple viral infections including herpes simplex virus and varicella zoster virus [91].

Several limitations may have influenced the obtained results. To begin with, the population of studies is mostly locally selected with limited number of patients, restricting the manifestations of the disease to a specific age or race. This factor makes it impossible to generalized the results of studies to a global scope.

Another source of error can be stated as a bounded literature due to the novelty of the virus which can generate problems like limited knowledge on the long-term effects of the disease, associated risk factors in patients and how they affect the overall neurological process and the association between the severity of the infection and how it can cause different levels of neurologic involvement. In addition, the relationship between the type of the treatment and the occurrence of neurological manifestations has not been addressed by many studies in the current literature.

A major source of unreliability was heterogeneity among diagnostic methods in different manifestations; for instance, anosmia was diagnosed mostly by self-reporting, but other methods of diagnosis were also mentioned among studies. In addition, many of the studies did not specify the stage of severity of the COVID-19 infection, which can greatly affect the type of the signs and symptoms that follow the infection.

Finally, the search strategy was designed using many keywords in several databases to ensure that all the related articles were included but considering the high rate in which the COVID-19 articles are being published, some sources of data might be missed.

## Conclusions

In conclusion, this study shows that neurological manifestations, most commonly general ones like headache and olfactory involvements, are an important aspect of the COVID-19 infection and the possibility of their occurrence should be considered in all infected patients. Therefore, our study underlines that all the COVID-19 patients should be carefully assessed and reassessed for detecting any neurological signs and symptoms. Considering how limited is known on the long-term effects of the neurologic involvement following this novel virus, continuous follow-up of infected patients is strongly suggested.

## Supplementary Information

**Additional file 1.** AMSTAR 2: a critical appraisal tool for systematic reviews that include randomised or nonrandomised studies of healthcare interventions, or both.

## Data Availability

All the data are available upon reasonable request.

## Abbreviations

COVID-19: Coronavirus disease 2019
AMSTAR-2: Assessment of Multiple Systematic Reviews-2
GBS: Guillen–Barré syndrome
PRISMA: Preferred Reporting Items for Systematic Reviews and Meta-analysis
URTI: Upper respiratory tract infection
IHC: Intracranial hemorrhage
SARS: Severe Acute Respiratory Syndrome
MERS: Middle East Respiratory Syndrome
